# The *NF1* Gene Contains Hotspots for L1 Endonuclease-Dependent *De Novo* Insertion

**DOI:** 10.1371/journal.pgen.1002371

**Published:** 2011-11-17

**Authors:** Katharina Wimmer, Tom Callens, Annekatrin Wernstedt, Ludwine Messiaen

**Affiliations:** 1 Division Human Genetics, Medical University Innsbruck, Innsbruck, Austria; 2 Medical Genomics Laboratory, Department of Genetics, University of Alabama at Birmingham, Birmingham, Alabama, United States of America; University of Pennsylvania, United States of America

## Abstract

Long interspersed (L1) and *Alu* elements are actively amplified in the human genome through retrotransposition of their RNA intermediates by the ∼100 still retrotranspositionally fully competent L1 elements. Retrotransposition can cause inherited disease if such an element is inserted near or within a functional gene. Using direct cDNA sequencing as the primary assay for comprehensive *NF1* mutation analysis, we uncovered in 18 unrelated index patients splicing alterations not readily explained at the genomic level by an underlying point-mutation or deletion. Improved PCR protocols avoiding allelic drop-out of the mutant alleles uncovered insertions of fourteen *Alu* elements, three L1 elements, and one poly(T) stretch to cause these splicing defects. Taken together, the 18 pathogenic L1 endonuclease-mediated *de novo* insertions represent the largest number of this type of mutations characterized in a single human gene. Our findings show that retrotransposon insertions account for as many as ∼0.4% of all *NF1* mutations. Since altered splicing was the main effect of the inserted elements, the current finding was facilitated by the use of RNA–based mutation analysis protocols, resulting in improved detection compared to gDNA–based approaches. Six different insertions clustered in a relatively small 1.5-kb region (*NF1* exons 21(16)–23(18)) within the 280-kb *NF1* gene. Furthermore, three different specific integration sites, one of them located in this cluster region, were each used twice, i.e. NM_000267.3(NF1):c.1642-1_1642 in intron 14(10c), NM_000267.3(NF1):c.2835_2836 in exon 21(16), and NM_000267.3(NF1):c.4319_4320 in exon 33(25). Identification of three loci that each served twice as integration site for independent retrotransposition events as well as 1.5-kb cluster region harboring six independent insertions supports the notion of non-random insertion of retrotransposons in the human genome. Currently, little is known about which features make sites particularly vulnerable to L1 EN-mediated insertions. The here identified integration sites may serve to elucidate these features in future studies.

## Author Summary

Repetitive retrotransposable elements, including LINE1 and *Alu* elements accounting for more than one fourth of the human genome, are still actively amplifying. It is widely believed that retroelements insert randomly in the genome. Retroelements newly inserted in the germ line may cause genetic disease, if a functional gene is disrupted. Up to now, only ∼65 well-characterized pathogenic retroelement insertions in 31 different human genes have been reported. Therefore, retrotransposition is suspected to be underdiagnosed as disease-causing mutation mechanism. Reporting 18 novel insertions in the *NF1* gene, all identified by a comprehensive RNA–based mutation analysis protocol, we show that L1 and *Alu* insertions represent 0.4% of all *NF1* mutations. Strikingly, we found three integration sites within this 280-kb gene that were used twice independently to insert a retroelement. One of these sites was located in a 1.5-kb “hotspot” region where four additional integration sites clustered. These findings, together with three additional integration sites used multiple times independently to insert retroelements in other genes, indicate that some genomic sites may be especially prone to host newly retrotransposed elements. As some of these sites are embedded in “hotspot” regions, larger flanking sequences may play a role in making these sites particularly vulnerable.

## Introduction

Long interspersed nuclear elements (LINE-1 or L1 elements) and *Alu* sequences belonging to the family of short interspersed nuclear elements (SINEs) still actively amplify in the human genome, by a process called retrotransposition. L1 elements comprise ∼17% of the human genome sequence [1] but of the ∼500.000 L1 copies only ∼80–100 are still fully capable of active retrotransposition [2]. Equally, only a small minority of the >1 million *Alu* elements comprising more than 10% of the human genome can retrotranspose in a non-autonomous process, using proteins encoded by L1 elements to mediate their mobility [3]. The active *Alu* elements are named *master* or *source Alu* elements [4]–[5]. L1 elements are transcribed by RNA polymerase II whereas polymerase III transcribes *Alu* elements. Both elements are transcribed from an internal promoter [6]–[7]. While L1 transcripts are polyadenylated after transcription, the poly(A) tail of *Alu* transcripts may be encoded directly from the genomic site of transcription [8]. *Alu* transcripts are then terminated at the 3′ end with a short run of U's [8]. L1 elements are autonomous retrotransposons. Active L1 elements are typically 6 kb in length and contain two non-overlapping open reading frames ORF1 and ORF2 [9]–[10]. The latter encodes a protein with endonuclease (L1 EN) and reverse transcriptase (L1 RT) activities [11]–[12]. It is generally accepted that L1 EN forms a nick at the insertion site of L1 elements and the L1 transcripts are reverse transcribed using the 3′ overhang of the nick as a primer [11]. The consensus cleavage site of L1 EN 3′-AA/TTTT-5′ (and derivates thereof) [11], [13] which usually cleaves at the bottom strand allows the T's at the 3′ terminus of the nick to prime reverse transcription from the poly(A) end of a L1 transcript. There is evidence that *Alu* elements are reverse transcribed by the same process called “target primed reverse transcription” (TPRT), but they need to “borrow” the factors for TPRT from L1 elements [14] and are, hence, called non-autonomous retrotransposons. Integration of the generated cDNA is less well understood. Generally, cleavage of the second DNA strand occurs a few base pairs (typically 7–20 bp) downstream of the first nick, causing target site duplications (TSD) and the free 3′ end at the second cleavage site is used to prime the second strand cDNA synthesis. The whole process results in the formation of a new DNA copy of an L1 or *Alu* element including the poly(A) tail flanked by short direct repeats of the duplicated target site. For a detailed description of the mechanisms of autonomous and non-autonomous retrotransposition see [8], [15] and papers cited therein.

Although retrotransposable elements have no immediate function in the cell, their motility can be important for genome plasticity and the creation of genetic variation [16]. Most recent studies [17]–[20] demonstrate that L1 and *Alu* elements dimorphic with respect to presence/absence at a given site contribute significantly to structural variation of the human genome. Furthermore, these studies show that current activity of mobile elements is likely to be higher than previously appreciated.

L1 and *Alu* elements are believed to insert randomly into the human genome. Hence, *de novo* transposition of an element may occasionally cause an inherited disease when the element is inserted into (or at proximity of) the coding region of a functional gene [21]. To date, only ∼65 cases of *de novo* L1 EN-mediated insertions of retrotransposable elements causing genetic diseases have been reported [22]–[23]. A systematic analysis of retrotranspositional events causing genetic disorders lists 48 such mutations consisting of 26 *Alu*, 15 L1, four SINE/VNTR/Alu (SVA) composite element insertions as well as three simple poly(A) insertions [24]. These *de novo* insertions were found in 31 different genes including one insertion in the *NF1* gene [25]. It has long been suspected that L1 EN-mediated retrotranspositional events are underreported as disease causing mutations, since they may be overlooked by the most commonly used mutation detection methods that rely on PCR amplification of small amplicons, i.e. exons from genomic DNA [26] (and reviewed later on in [27]–[28]). Here we report 18 novel L1 EN-mediated insertions in the *NF1* gene: one poly(T), three L1 and 14 *Alu* insertions were all identified using an RNA-based core assay as the starting point for comprehensive *NF1*-gene analysis illustrating the strength of this approach in identifying also this complex type of mutations. Of note, six of the integration sites were located within a relatively small genomic region of 1500 bp between *NF1* exons 21 (16) and 23 (18) (exons are numbered consecutively according to the NCBI reference sequence NM_000267.3; in addition, the more widely known historical legacy numbers, originally designated by the international *NF1* consortium, are given in parentheses). Even more striking, three of the integration sites within the *NF1* gene were used twice. Our results indicate and confirm that some genomic locations may be especially prone to L1 EN mediated retrotransposition.

## Results

### Identification of 18 L1 EN-dependent *de novo* insertions into the *NF1* gene

**Figure 1:**
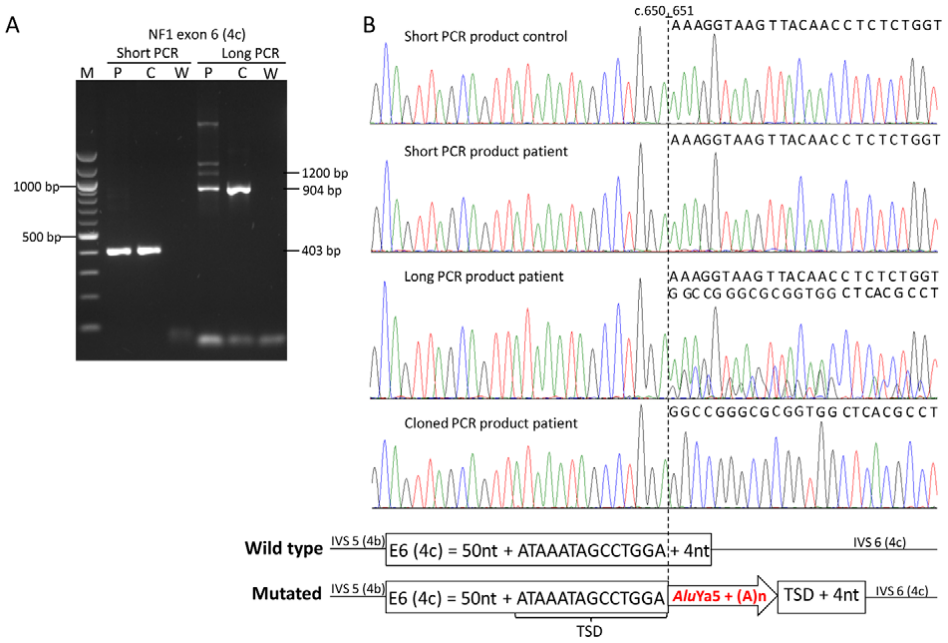
Example of *de novo Alu* insertion detection by improved PCR conditions. A) Agarose gel with PCR products of exon 6 (4c) generated with two different primer pairs and PCR conditions generating a 403-bp (short) and a 904-bp (long) wild type PCR product (see Table S1). PCR products were generated from a control individual (C) and the patient (P) harboring an *Alu*Ya5 insertion in exon 6 (4c) (W = water control). Amplification of the large PCR product from the patient revealed the presence of extra bands of increased size, i.e. a ∼1200-bp band representing the homoduplex of the PCR product from the mutant allele and two larger bands probably derived from heteroduplexes of this PCR product with the PCR product from the wild type allele, which are neither present in the control nor in the shorter amplified PCR products. (B) Sequences of the short and long PCR products from the control individual and the patient; the sequence of the long PCR product but not of the short PCR product of the patient shows starting at nucleotide position c.651 (vertical dotted line) a background sequence coming from the inserted *Alu*Ya5 element (see scheme below the sequences). The sequence of a cloned long PCR product clearly shows the presence of the *Alu*Ya5 element within the *NF1* exon 6 (4c).

Direct cDNA sequencing used as the core assay of comprehensive *NF1* mutation analysis in the two centers (UAB and MUI) uncovered heterozygous (∼50% of the transcripts affected) splicing alterations in 18 NF1 index patients (Table 1) that could neither be explained by a heterozygous mutation of the splice regulatory elements of the affected exon and/or the flanking intronic sequences nor by a deletion of the genomic DNA. However, fragment analysis of the gDNA amplicons of the affected exons showed in some cases a faint extra band of larger size absent in the PCR products of a control DNA (Figure S2A). Sequence analysis of these PCR products revealed a faint additional sequence, besides the wild type sequence, starting at the position where the retroelement was inserted (Figure S2B). Manual reading of the background sequences revealed the presence of *Alu* elements inserted within the exonic sequences. In addition to the approximately 280-bp long *Alu* elements a 60–120-bp long poly(A) tail -or a poly(T) tail- was inserted depending on whether the *Alu* element was inserted in the sense or antisense orientation with respect to the *NF1* reading frame (Figure S2B). As expected for *de novo* retrotransposed *Alu* elements the inserted sequences were flanked by short duplicated sequences derived from the insertion sites, i.e. target site duplication (TSD). We reasoned that the substantial increase in size of the mutant exons containing the *Alu*/L1 insertions, compared to the wild type exons caused allelic drop-out under standard PCR conditions. We increased the extension time of the PCR reactions and/or increased the size of the PCR amplicons to enhance the amplification of the larger mutant alleles and, hence, facilitate the detection of possible *de novo Alu* insertions. Amplification of all exons with so far unexplained splicing alterations using PCR conditions optimized to amplify larger PCR products (Figure 1A and 1B) led to the identification of 14 different *de novo Alu* insertions (Table 1) as well as an approximately 120-bp poly(T) stretch in exon 25 (19b) resulting in skipping of the last 40 nucleotides of this exon in the mRNA transcripts.

**Table 1:** List of all *Alu*, L1 and poly(T) insertions uncovered in the *NF1* gene.

No.	Patient no.	Location of insertion	Inserted element (*Alu* family)	*Alu*/L1 element size (bp)/5′ truncation (Y/N)	Orientation of the insert with regard to NF1 reading frame	length of poly (A) tail (bp)	Mutation according HGVS nomenclature	Effect on mRNA	splice type as depicted in figure 3
1	UAB-R10408	IVS 14 (10c)	*Alu*Y	280/N	S	no estimate	c.1642-1_1642insAluY,1642-11_1642-1dupAAAAAATTCAG	r.1642_1721delIF skip E15 (E11)	1
2	UAB-R119201	IVS 10 (8)	*Alu*Y	281/N	AS	no estimate	c.1186-86_1186-16delinsAluY	r.1186_1260del IF skip E11 (9)	1
3	MUI-1	E6 (4c)	*Alu*Ya5	282/N	S	∼60 bp	c.650_651dupinsAluYa5, 637_650dupATAAATAGCCTGGA	r.587_654delIF skip E6 (E4c)	2
4	UAB-R37305	E21 (16)	*Alu*Ya5	284/N	AS	120 bp	c.2835_2836insAluYa5, 2824_2835dupAGCAAGTTTTTT	r.2618_2850delskip last 233 nt E21 (E16)	3
5	UAB-R37616	E21 (16)	*Alu*Ya5	281/N	AS	no estimate	c.2835_2836insAluYa5, 2819_2835dupCCATCAGCAAGTTTTTT	r.2618_2850delskip last 233 nt E21 (E16)	3
6	UAB-R30609	E22 (17)	*Alu*Ya5	284/N	AS	110 bp	c.2858_2859insAluYa5,2851-7_2858dupTCTTTAGGTTTTA	r.2851_2990delOOF skip E22 (E17)	2
7	UAB-R81017	E33 (25)	*Alu*Ya5	279/N	S	no estimate	c.4319_4320insAluYa5,4305_4319dupAAAAGAAGAACATAT	r.4270_4367delOOF skip E33 (E25)	2
8	MUI-2	E47 (38)	*Alu*Ya5	264/Y	AS	60–85 bp	c.6951_6952ins AluYa5,6936_6951dupAGGTACCGCACTTCTT	r.[6859_6999del;6938_6999del]IF skip E47 (E38); skip last 62 nt of E47 (E38)	2&3
9	UAB-R869001	E12 (10a)	*Alu*Yb8	249/Y	S	121 bp	c.1354_1355insAluYb8, dup1345_1354dupAAAGCAGTGC	r.1355_1392delins61skip of the last 38 nt E12 (E10a) & insertion of first 61 nt of the inserted Alu element	5
10	UAB-R164201	IVS 14 (10c)	*Alu*Yb8	288/N	AS	no estimate	c.1642-12_1642-11insAluYb8,1642-28_1642-12dupTTGTCTTTCTCTTTTTT	r.1642_1721del80OOF skip E15 (11)	1
11	UAB-R39428	E21 (16)	*Alu*Yb8	289/N	AS	120 bp	c.2439_2440insAluYb8, 2428_2439dupAAGACCATTGTT	r.2410_2638delskip first 229 nt of E21 (E16)	4
12	UAB-R50109	E22 (17)	*Alu*Yb8	288/N	S	78–178 bp	c.2979_2980insAluYb8, 2966_2979dupAAACAATGATGTTA	r.2851_2990delOOF skip E22 (E17)	2
13	UAB-R340101	E33 (25)	*Alu*Yb8	288/N	S	118 bp	c.4319_4320insAluYb8, 4305_4319dupAAAAGAAGAACATAT	r.4270_4367delOOF skip E33 (E25)	2
14	UAB-R07118	IVS48 (39)	*Alu*Yb8	268/Y	AS	121 bp	c.7127-5_7127_4insAluYb8,7127-20_7127-5dupTGTTTGTTTGTTTTTT	r.7127_7258delIF skip E49 (40)	1
15	UAB-R75103	E25 (19b)	poly(T) strech	∼130/Y	AS	120 bp	c.3312_3313insT_(n∼120)_,3307_3312dupTTTCTT	r.3275_3314delskip last 40 nt of E25 (E19b)	3
16	UAB-R316001	E23 (18)	L1(preTa)	∼1800/Y	S	no estimate	c.3048_3049insTGTGAATTinsL1(Ta)_0rf2, 3033_3048dupAAAAACGAAACTGTGT	r.2991_3113delIF skip E23 (E18)	2
17	UAB-R01429	E39 (30)	L1(Ta)	∼6000/N	S	no estimate	c.5606_5607insL1, 5594_5606dupTAAAAATCGAGGG	r.5607_5749delins96 skip of the last 143 nt of E39 (30) & insertion of first 96 nt of the inserted L1 element	5
18	UAB-R91409	IVS9 (7)	n.k.	∼2200/Y	AS	no estimate	c.1062+195_1062+196insL1, 1062+185_1062+195dupTTCTTTTACCA	r.889_1062delins130OOF skip E9 (7) & ins 130 nts of L1 element	6

Abbreviations: IF = is in frame; OOF = out of frame; E = exon; IVS = intron; Y = yes; N = no; S = sense; AS = antisense; n.k. = not known; nt = nucleotides.

To further confirm the presence of the *Alu* elements within the mutant *NF1* alleles and to determine precisely the inserted sequence, the PCR products showing the extra bands of increased size were cloned and sequenced (Figure 1B). Additionally or alternatively, *Alu*-insertion-specific primers were used together with the regular exon primer at the opposite site of the exon to specifically amplify and subsequently sequence the mutant alleles (Figure S3). All 14 *Alu* insertions identified are listed in Table 1. Alignment of the inserted *Alu* sequences with the consensus sequence of the different *Alu* families (as deposited in Repbase Giri [29]) showed that two *Alu* elements belong to the *Alu*Y, six to the *Alu*Ya5 and six to the *Alu*Yb8 family (Figure S4). Three of the *Alu* insertions were truncated and lacked at their 5′ end 17, 20 and 39-bp, respectively, (see patients MUI-2, UAB-R07118 and UAB-R869001 in Figure S4). Six of the *Alu* elements were inserted in sense and eight in anti-sense orientation.

**Figure 2:**
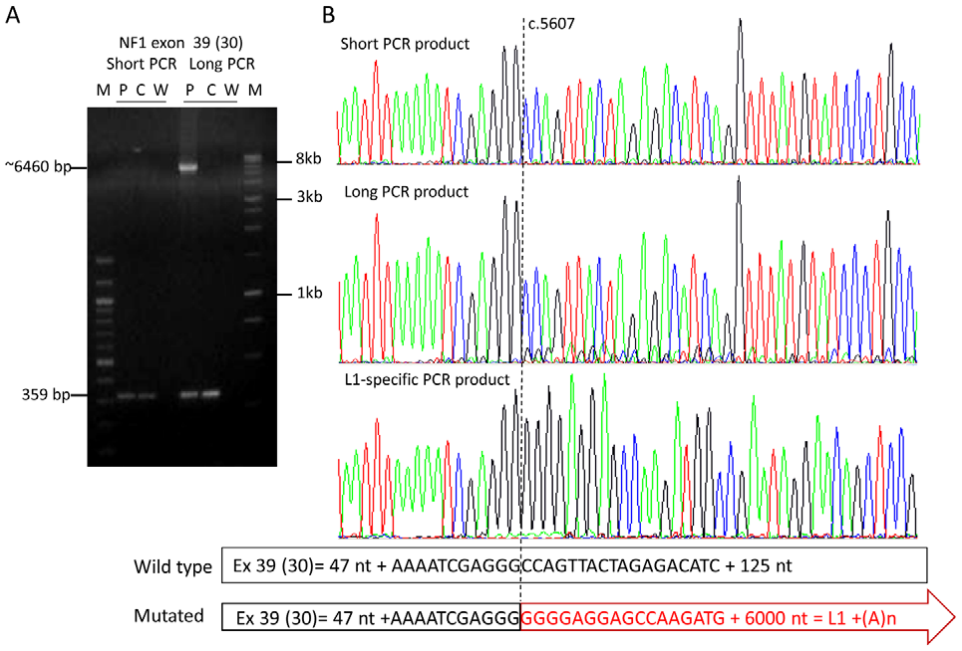
Amplification of the mutant allele containing a full-length L1 element inserted into exon 39 (30). (A) Agarose gel with PCR products of exon 39 (30) generated with two different PCR conditions. PCR products were generated from a control individual (C) and the patient (P) harboring a full-length L1 element in exon 39 (30) (W = water control). The PCR conditions (short) used in the diagnostic laboratory generate only the 359-bp product from the wild type allele present in the control and the patient, whereas a >6000-bp PCR product derived from the mutant allele was amplified along with the wild type 359-bp PCR product using the Expand Long Template PCR system kit (Roche), buffer system 2 and 3 (long) (see Table S1). (B) Sequences of the PCR products from the patient generated with the PCR conditions “short” and “long”; the sequence of the long PCR product but not of the short PCR product of the patient shows starting at nucleotide position c.5607 (vertical dotted line), besides the main wild type sequence, minor traces of a sequence coming from the inserted L1 element (see scheme below the sequences). The presence of the L1 element within the *NF1* exon 39 (30) was confirmed using a L1-specific PCR (primers: 39f and L1-5_39r). The entire 6021-bp sequence of this L1 (Ta-1d) element is deposited in Figure S5.

Increase of the amplicon size and the PCR extension time of exons 39 (30), 23 (18) and 9 (7) did not allow resolution of the cause of their mis-splicing in the patients UAB-R01429, UAB-R316001 and UAB-R91409, respectively. Use of a Taq polymerase enabling amplification of extremely long PCR products (up to >6 kb) was needed to amplify the mutant exons which had 10- and 17-times increased in size compared to their wild type normal allele in patients UAB-R316001 and UAB-R01429, respectively. A 6-kb full-length L1 element inserted in sense orientation into exon 39 (30) was identified in patient UAB-R01429 (Figure 2A). Full sequencing of the inserted L1 element showed it to belong to the youngest L1 (Ta-1d) subset since it carried all sequence variants that distinguish this L1 (Ta) group from others [2], [30]. It contained 10 deviations from the consensus sequence of hot L1 sequences as given by [2] (Figure S5). Sequence alignment with all known intact L1s of the Ta-1d subset [31] showed that the closest related hot L1 (Ta-1d) element is contained in NCBI sequence AL356438. Only six nucleotides differentiate the full-length L1 identified in exon 39 (30) from this possible precursor sequence. A 5′-truncated L1 element was found to be inserted in sense into exon 23 (18) in patient UAB-R316001. The latter sequence contained 1753 bp of the 3′-half of ORF2 and the poly(A) tail but lacked the ORF1 and the promoter region of L1 elements. Furthermore, it was preceded by a short 8-bp sequence neither derived from the target sequence nor from the inserted L1 element, a finding that previously has been observed for L1 retrotransposition events in cultured cells [32]–[34]. This L1 element belongs to the L1(pre-Ta) subset since it carries a 3-bp ACG at the site discriminating the L1(Ta) and L1(pre-Ta) subsets [35]. In intron 9 (7) we found a sequence inserted that contained a poly(T) stretch at the 5′-end indicating that the poly(A) tail of the inserted retrotransposon transcript had annealed to the sense strand where reverse transcription from the template started. Sequencing from the reverse strand showed, however, that the 3′-end of the inserted sequence contained part of an L1 ORF2 sequence (see Figure S6). This sequence was inserted in sense orientation with regard to the *NF1* coding sequence suggesting that during the process of retrotransposition the orientation of the reverse transcription from the L1-RNA template that started at the sense strand of the *NF1* gene switched and continued from the anti-sense strand. Due to experimental difficulties and lack of sufficient patient's DNA it was not possible to assess the full sequence of the inserted L1 element and, therefore, the breakpoint of inversion could not be defined.

### Analysis of insertion sites

Alignment of the sequences at the integration sites (Table 2) shows that all integration sites match the reported L1 EN consensus cleavage site [13], indicating that all insertions arose via L1 EN-mediated retrotransposition. The exact insertion site of an *Alu*Y element into intron 10 (8) cannot be determined due to a 71-bp deletion associated with this insertion that causes also lack of a TSD. Nevertheless, the most likely insertion site matching a L1 EN cleavage site is tentatively given in Table 2. The alignment of the integration sites also shows that the 18 retroelements inserted in 15 different integration sites with three of them used twice independently. We found in two non-related patients (UAB-R10408 and UAB-R164201 in Table 1) an *Alu*Y and *Alu*Yb8 element inserted into the splice acceptor site of intron 14 (10c). The same L1 EN cleavage site was used once at the anti-sense strand to insert the *Alu*Y element in sense orientation with respect to the coding sequence of the *NF1* gene (integration site according to HGVS nomenclature c.1642-1_1642) and in an unrelated patient once at the sense strand to insert the *Alu*Yb8 element in anti-sense orientation (integration site according HGVS nomenclature c.1642-12_1642-11). Furthermore, we found in two non-related patients (UAB-R81017 and UAB-R340101 in Table 1) an *Alu*Ya5 and an *Alu*Yb8 element, respectively, inserted at the identical integration site, c.4319_4320, in exon 33 (25). Both *Alu* elements were inserted in sense orientation and flanked by the same sized TSD (Table 2). Finally, in two non-related patients (UAB-R37616 and UAB-R37305 in Table 1) -one of them being a sporadic patient proven to have a *de novo* insertion- an *Alu*Ya5 element inserted at the identical position, c.2835_2836, in exon 21 (16). The length of TSDs flanking the two insertions differed between both patients (Table 2) confirming further that two independent events led to the formation of these two mutant alleles. This site was located within a 1.5-kb genomic region containing exons 21 (16), 22 (17) and 23 (18) that harbor a total of six independent retrotranspositions. The remaining eight integration sites of *de novo* insertions were distributed over the entire *NF1* gene from exon 6 (4c) to intron 48 (39).

**Table 2:** Sequences at the integration sites aligned to the L1 endonuclease cleavage consensus site.

Integration sites	TSD	Orientation	*Alu* family	*NF1* location
3′AAAGAGAAAAAA TTTTTTAAGTCCGAGACGACCAAG 5′ ^§^	11	sense	Y	I 14 (10c)
3′TTGTCGTTTATC TTTCAAATTTTTTTGTGATTCAAA 5′ *	-	antisense	Y	I 10 (8)
3′GTCAATCGTCAA TATTTATCGGACCTTTTCCATTCA 5′	14	sense	Ya5	E 6 (4c)
3′AGGAACCCTCAG TTTTTTGAACGACTACCATAAGAA 5′ ^#^	12	antisense	Ya5	E 21 (16)
3′AGGAACCCTCAG TTTTTTGAACGACTACCATAAGAA 5′ ^#^	17	antisense	Ya5	E 21 (16)
3′CCATAGTCAGTT ATTTTGGATTTCTTTCTTGTTTAT 5′	13	antisense	Ya5	E 22 (17)
3′ACAAGAGAAGTG TTTTCTTCTTGTATACGCCGGAAA 5′ ^‡^	15	sense	Ya5	E 33 (25)
3′GTCCAAAACAAG TTCTTCACGCCATGGACGACTTAT 5′	16	antisense	Ya5	E 47 (38)
3′CTTTGTGAAGTA TTTCGTCACGTTCCAACACCTCGT 5′	10	sense	Yb8	E 12 (10a)
3′GGACTTAAAAAA TTTTTTCTCTTTCTGTTCCGGCCC 5′ ^§^	17	antisense	Yb8	I 14 (10c)
3′GTAAGCGGAGAA TTGTTACCAGAACACTTCCGAAAG 5′	12	antisense	Yb8	E 21 (16)
3′TTCGATCGTAAC TTTGTTACTACAATTTAGACCAGT 5′	14	sense	Yb8	E 22 (17)
3′ACAAGAGAAGTG TTTTCTTCTTGTATACGCCGGAAA 5′ ^‡^	15	sense	Yb8	E 33 (25)
3′GGACATGGGATG TTTTTTGTTTGTTTGTTTGTTTGT 5′	16	antisense	Yb8	I 48 (39)
3′ACGTTAAGTTTA TTTTTGCTTTGACACAGTTAATCA 5′	16	sense	L1P1_orf2	E 23 (18)
3′CATGGAAATTAA ATTTTTAGCTCCCGGTCAATGATC 5′	13	sense	LINE 1	E 39 (30)
3′AATACGAATAAA TTCTTTTACCAAGTAACATCTAAG 5′ 3′ACTTTAAATGAA TTCTTTATTGACACTAAACCGAAG 5′	116	antisenseantisense	L1T_(n∼120)_	I 9 (7)E 25 (19b)

AA-TTTT L1 endonuclease cleavage consensus site.

*This integration site cannot be unequivocally determined due to an associated 71-bp deletion and the lack of a TSD. The given integration site assumes either a model where the second nick occurred downstream instead of upstream to the first nick that affected the *NF1* sense strand or a model where the reverse transcribed *Alu* cDNA strand (termed minus strand) invaded a double-strand break 71 bp downstream of the given integration site. Integration sites indicated with the same symbole §, # or ‡ are identical.

### Different splicing effects are associated with *de novo Alu* and L1 insertions

**Figure 3:**
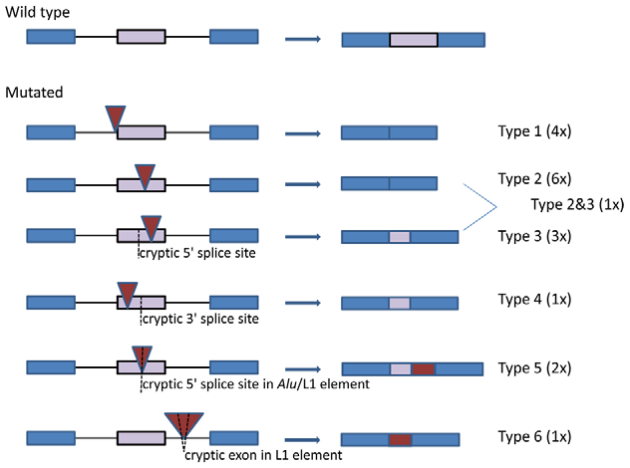
Schematic presentation of the splicing effects caused by the identified *Alu* and L1 insertions. The affected exon is indicated with a purple bar and the two flanking exons with blue bars. Intronic sequences are indicated by black lines. Red triangles denote the inserted *Alu* and L1 sequences and cryptic splice sites within an exon or the retrotransposon are indicated by a dotted line. The genomic sequence and the ensuing mRNA transcripts are depicted at the left and right hand site, respectively.

Four out of 14 *Alu* elements (UAB-R10408, UAB-R164201, UAB-R07118 and UAB-R119201 in Table 1) were inserted into the canonical AG-dinucleotide (1/4) or the polypyrimidine tract (3/4) of the 3′ splice sites of introns 14 (10c), 48 (39), and 10 (8), respectively. Disruption of these splice sites readily explains the observed skipping of the downstream exons (type 1 splicing effect in Figure 3). Skipping of the affected exon was also observed in five other patients with *Alu* insertions, i.e. two insertions at the identical site in exon 33 (25), two insertions at different integration sites in exon 22 (17) and one insertion in exon 6 (4c) (type 2 effect in Figure 3). Two *Alu*Ya5 elements (UAB-R37305 and UAB-R37616 in Table 1) inserted at the identical integration site c.2835_2836 in exon 21 (16) in two unrelated patients causing skipping of the last 233 nucleotides of this 441-bp exon. This partial exon skipping results from the use of a cryptic 5′ splice site located upstream of the integration site (type 3 effect in Figure 3). Similarly, integration of an *Alu*Yb8 element at position c.2439_2440 in the 5′ half of exon 21 (16), 30 nucleotides downstream of the intron-exon 21 (16) border, resulted in the use of a cryptic 3′ splice site downstream of the integration site and loss of the first 229 bp of the exon 21 (16) (mutation UAB-R39428 in Table 1; type 4 effect in Figure 3). Insertion of the approximately 120-bp poly(T) stretch at c.3312_3313 in exon 25 (19b), 3 nucleotides upstream of the exon-intron border also led to use of a cryptic 5′ splice site and loss of the last 40 nucleotides of this exon (mutation UAB-R75103; type 3 effect in Figure 3). Insertion of an *Alu*Ya5 element into exon 47 (38) resulted in at least two different splicing effects, i.e. skipping of the exon and use of an exonic cryptic 5′ splice site upstream of the integration site (mutation MUI-2; type 2 and 3 splicing effect in Figure 3; see also Figure S1). Insertion of an *Alu*Yb8 element into exon 12 (10a) also resulted in a more complex splicing defect. This element lacks the first 39 bp at its 5′ end (UAB-R869001 in Table 1 and Figure S4). A cryptic 5′ splice site within this truncated *Alu* element is used instead of the natural one of the exon 12 (10a), leading to transcripts containing the first 61 nucleotides of this *Alu* element but lacking the 3′ half of exon 12 (10a), downstream of the *Alu* integration site (type 5 effect in Figure 3).

Insertion of the 5′-truncated L1 element into exon 23 (18) also caused simple exon skipping in the transcripts of the patient UAB-R316001 (type 2 splicing effect in Figure 3). The insertion of the 5′-inverted L1 element 195 nucleotides downstream of exon 9 (7) caused skipping of exon 9 (7) as well as inclusion of a 130-bp cryptic exon embedded within the inserted L1 element (patient UAB-R91409; type 6 effect in Figure 3; see also Figure S6). Finally, a 5′ splice site within the full-length L1 element inserted into exon 39 (30) is used instead of the natural 5′ splice site of exon 39 (30), leading to a transcript lacking the 143 nucleotides of the exon downstream of the integration site and containing instead the first 96 nucleotides of the L1 element (patient UAB-R01429; type 5 effect in Figure 3).

Taken together, all *Alu* and L1 elements as well as the insertion of a poly(T) element lead to altered splicing of the transcripts from the mutant allele. None of the inserted *Alu* or L1 elements was fully included into the transcripts.

## Discussion

Here we report the identification of 18 pathogenic L1 EN-mediated insertions into the *NF1* gene. This is the largest number of disease causing mutations of this type found in a single human gene. Sixteen of these insertions were found in a cohort of 4300 unrelated *NF1* mutation-positive patients analyzed at UAB and two in a cohort of 200 unrelated *NF1* mutation-positive patients analyzed at MUI. Taken together, this suggests that retrotransposon insertions account for ∼0.4% (18/4500) of *NF1* mutations identified. This frequency is two to four times higher than previously anticipated [36] and expected from a database survey in human genetic diseases [28]. Two mutually non-exclusive explanations for this observation may be taken in consideration.

### Improved detection of L1 EN-mediated *de novo* insertions by *NF1* cDNA sequencing

Firstly, mutations due to retrotransposition have long been thought to be underestimated, since they may be missed by mutation detection methods relying on PCR amplification of small amplicons, i.e. exons, from genomic DNA [26]–[28] and references cited therein. Indeed, all insertions reported here were identified because they altered splicing of the transcripts as detected by direct cDNA sequencing [37]. In order to identify the cause of the splicing defects, i.e. the *Alu*, L1 and poly(T) stretch insertions, it was necessary in most of the cases to modify the PCR conditions to prevent allelic drop-out of the substantially larger mutant allele. Hence, most of these mutant alleles would be missed by an exon by exon-sequencing approach. Similar findings were reported for the *BRCA1* and *BRCA2* genes [38] and even for genes involved in X-linked diseases [39] although allelic drop-out is expected to be less severe in X-linked diseases due to hemizygosity of the mutant allele in the affected males. Thus, our results demonstrate that RNA-based mutation analysis protocols are more apt to uncover L1 EN-mediated retrotransposon insertions.

### The *NF1* gene may be a preferred target of retrotransposon integration in the human genome

Secondly, it is possible that the *NF1* gene is particularly susceptible to retrotransposon integration. It is striking that three specific sites within the *NF1* exons 21 (16) and 33 (25) and the splice acceptor site of intron 14 (10c) hosted each two independently retrotransposed elements. Moreover, one of these sites is located in a small region of approximately 1.5 kb within the ∼280-kb *NF1* gene where six of the 18 insertions cluster. Together, these finding strongly support the notion of an non-random *de novo* insertion of retrotransposons in the human genome [24]. A systematic analysis of 48 previously reported simple L1 EN-dependent insertions showed that some genes, e.g. *F8* and *F9* mutated in hemophilia B, may have hotspot regions for retrotransposon integration [24]. Furthermore, three integration sites in three other genes, i.e *APC*, *F9* and *BTK*, have been reported to be used twice independently for L1 EN-mediated retrotransposon insertions [24], [39]. Taken together with our findings in the *NF1* gene, a tenth (6/66) of the well characterized sites that harbor disease causing retrotransposon insertions have been used multiple times with half of them identified in the current study. It remains to be elucidated which features make these sites particularly vulnerable to L1 EN-mediated insertions. The fact that some of these sites are embedded in a larger sequence context that appears to be a hotspot region for insertions may indicate that flanking sequences and possibly also the chromatin structure in these regions may play a role.

It has been suggested that *Alu* and L1 elements have a similar retrotransposition efficiency via-à-vis the molecular retrotransposition machinery [40]. However, as in the *NF1* gene also in previously published disease causing retrotranspositional insertions a preponderance of *Alu* over L1 insertions can be observed. As a possible explanation Dewannieux et al. [3] propose that L1 elements harboring a disrupted ORF1, but retaining a functional ORF2, should still be competent for *trans* mobilization of *Alu* elements even if they are unable to promote their own transposition. In other words, there appear to be more potential “drivers” for *Alu* retrotransposition than there are for L1 retrotransposition [4] potentially explaining the preponderance of disease-causing *Alu* over L1 insertions [24].

### 18 novel *Alu*/L1 insertions substantially increase the number of known disease-causing mutations of this type

With this report we substantially increase the number of known disease causing *Alu*, L1 and simple poly(T) insertions. Hence, our data confirm and extend several observations deduced from 48 previously analyzed pathological insertions [24]. All inserted retrotransposons in our study contained a poly(A)/poly(T) tail with a size in the range from 60–178 bp and all but one were flanked by TSDs with a size in the range of 6–17 bp (see Table 1 and Table 2). All integration sites (Table 2) matched the previously reported consensus sequence of the L1 EN cleavage site [13], [41] and there was no preferential orientation of the inserts with respect to the *NF1* coding sequence. All *Alu* insertions belong to the evolutionary youngest “Y” *Alu* family (see Figure S4). Three of them (MUI-2, UAB-R07118 and UAB-R869001 in Table 1) lacked 17–39 bp at their 5′ end when compared to the *Alu* consensus sequence (Figure S4). According to [42] these three elements would fall into the Group II *Alu* inserts which represent ∼8% of all *Alu* elements polymorphic with respect to presence or absence in the human genome. The poly(T) sequence inserted into the *NF1* exon 25 (19b) may represent a severely truncated Group III *Alu* insertion [42]. Equally, this poly(T) stretch may result from a severely truncated L1 element or even a processed pseudogene. One *Alu*Y element inserted into intron 10 (8) was accompanied by a 71-bp deletion and lacked a TSD. Different possible mechanisms for the loss of genomic sequences in association with EN-dependent retroelement insertions have been discussed [24]. One suggested mechanism depicted in Figure 6A in [33] assumes that the second-strand nick at the top strand of the L1 target site is made a few bp to the “left” of the initial nick on the bottom strand rather than to the “right” causing the loss of a few bp at the insertion site. A model that would explain the larger deletions of several kb assumes that the reverse-transcribed cDNA strand of the retroelement is involved in double-strand break processing and invades a double-strand break to the “left” of the first-strand nick (see Figure 6B in [33]). Both models may theoretically apply to the 71-bp deletion associated with the *Alu*Y insertion into *NF1* intron 10 (8). In both models the first-strand L1 EN-mediated nick would have occurred at position c.1186-85_1186-86 in intron 10 (8) (as tentatively given in Table 1 and Table 2). However, it is also possible that an EN-independent integration mechanism as previously observed in certain Chinese hamster ovary (CHO) cell models [41], [43] has led to the TSD-lacking and deletion-associated integration of this *Alu*Y element.

According to Kojima [44] L1 elements of the human genome can be classified into three categories: full-length, 5′-truncated and 5′-inverted, comprising respectively 25.5%, 43.8% and 30.7% of all L1 elements. Here we found an example of each category. The full-length element belonging to the youngest L1(Ta-1d) subset was a simple insertion in sense orientation with regard to the *NF1* coding sequence flanked by a 12-bp TSD. The 5′-truncated element lacked the promoter region, the entire ORF1 and the first 2080 bp of the 3825-bp ORF2. Of note, this is only the second reported pathogenic insertion of an older and less active L1(pre-Ta) element [2]; the first was found in the *F8* gene in a hemophilia A patient [21]. The 5′ inverted L1 element contained a poly(T) tail at the 5′ end but the analyzed sequence at the 3′ end was inserted in sense orientation and contained 1088 bp from the center of the 3825-bp ORF2 (Figure S6). This suggests that during the process of retrotransposition the orientation of the reverse transcription from the L1-RNA template that started at the sense strand of the *NF1* gene switched and continued from the anti-sense strand. The presence of a 2-bp micro-homology that may have promoted strand switching due to secondary binding between the RNA-template and the 3′-end of the 5′-overhang at the antisense strand supports the model of twin priming [45] (depicted also in Figure 1 in [44]) as the underlying mechanism leading to the insertion of this 5′ inverted L1 element.

### Splicing defects are the prevalent effect of L1 EN-mediated retroelement insertions

Only four of the *Alu* insertions identified here directly affect splice sites. Nevertheless, all L1 EN-mediated insertions in this study affected *NF1* splicing in the patients. The predominant effect of the 13 exonic *Alu*, L1 and poly(T) insertions was exon skipping (6/13 cases) or use of a cryptic splice site -either a cryptic 5′ splice site upstream (3/13 cases) or a cryptic 3′ splice site downstream of the integration site (1/13 cases). In one of 13 cases both of these splice effects were observed in the same patient. In only two cases part of the *Alu* or L1 element were contained in the mRNA transcripts, inserted into exon 12 (10a) and 39 (30) respectively. Overall, we do not believe that ascertainment bias can explain why exon skipping and/or use of cryptic splice site are the main effects of the *de novo Alu* and L1 insertions in exonic sequences. Firstly, because we have no evidence that the long-range RT-PCR reactions used in the applied assays would miss *Alu* insertions in exons or introns that lead to full or partial exonisation of the inserted sequences in the mRNA transcripts. On the contrary, duplications of single or multiple exons which lead to a similar size increase of the RT-PCR products were readily detected by our assays [46]. Secondly, in agreement with our observations, exon skipping was also the reported effect of 5/7 L1 EN-mediated integrations for which RNA data were available in the literature (see Table 1 in [28]). Hence, following an exon definition model of splicing [47], our data indicate that the main effect of exonic *Alu* and L1 insertion is weakening of the exon definition resulting in altered splicing of the affected exon. Currently it is not fully evaluated by which mechanisms the inserted sequences reduce exon recognition by the splicing machinery. It has been proposed that inserted sequences may disrupt specific *cis*-acting exonic splice elements, such as exonic splicing enhancers (ESEs) (see [28] and references cited therein). However, it is unlikely this pertains to all or to the majority of exonic *de novo* insertions. Therefore, we favor the hypothesis that the insertion of a relatively large number of nucleotides (300–2000 bp) weakens exon definition simply by increasing the size of the affected exon and/or by disrupting the exonic structure of *cis* acting elements in a more general sense, e.g. by increasing the distance of exonic *cis* regulatory elements and the splice sites of the exon. In this respect it is of note that the use of cryptic 5′- and 3′- splice sites in exons 21 (16) and 25 (19b) is also observed in transcripts from mutant *NF1* alleles that carry single nucleotide alterations destroying the respective natural 5′- and 3′- splice sites of these exons ([48] and Messiaen unpublished results). This observation may indicate that some of the inserted sequences weaken particularly the downstream or upstream splice site while the definition of the respective other splice site is unaffected which in certain instances favors use of a cryptic splice over skipping of the affected exon [48]–[49].

The integration of an inverted L1 element into intron 9 (7) caused a complex splicing effect, i.e. insertion of a cryptic exon embedded in the inserted L1 element and skipping of the preceding exon 9 (7). A similar splicing defect was described in a chronic granulomatous disease patient who carried a truncated L1 element in intron 5 of the *CYBB* gene [50]. As in the *NF1* gene, the cryptic exon inserted in the transcripts of *CYBB* was not better defined by the splice sites compared to the flanking natural exons that were skipped in those transcripts containing the L1 derived cryptic exon. Hence, it remains to be explained why insertion of these L1 elements lead to exonisation of the cryptic exon on the account of the adjacent natural ones.

### Concluding remarks

Our results clearly show that RNA-based mutation analysis strategies have the potential to detect disease-causing L1 and *Alu* insertions. In addition, the RNA-based comprehensive *NF1* mutation detection approaches unambiguously identify and functionally characterize other classes of mutations usually missed by DNA-based mutation detection strategies, such as intronic alterations outside the canonical splice site dinucleotides (GT-AG) (including deep intronic mutations), as well as silent and missense mutations with an effect on splicing [48], [51]. Still this approach may underestimate L1 EN-mediated insertions. *De novo* retrotransposon insertions within the 3′- or 5′-UTR can lead to reduced expression by the disruption of gene regulatory elements [52]–[53]. Furthermore, insertion of L1 elements within introns has been shown to reduce mRNA transcript levels. This phenomenon is related to RNA polymerase II elongation defects and/or premature polyadenylation caused by the L1 elements [54]–[55]. Thus, it is possible that insertions especially in the regulatory or intronic regions of the gene may still be missed by the here applied *NF1* mutation analysis assay.

## Material and Methods

### Patients

All mutations reported were uncovered in samples from unrelated index patients sent for clinical *NF1* testing to two centers, i.e. the Medical Genomics Laboratory at the University of Alabama in Birmingham (UAB) and the Division of Human Genetics, Medical University Innsbruck (MUI). Informed consent was obtained from all patients. This study was approved by the ethical committee from both institutions.

### Identification of splicing defects by direct cDNA sequencing

In both laboratories the primary assay for comprehensive *NF1* mutation analysis is a direct cDNA sequencing approach that is based on the amplification of the entire *NF1* coding region in three (Birmingham, AL) or five (Innsbruck) overlapping RT-PCR fragments and subsequent sequencing of the entire PCR products with 18 (20) internal primers. Details on the RT-PCR reactions and primers can be found in [37], [56]. To avoid illegitimate splicing, known to lead to multiple aberrant splice variants that impede the detection of mutations in an RNA-based approach [51], [57], total RNA is extracted from phytohemagglutinin (PHA)-stimulated short-term lymphocyte cultures treated with 200 µg/ml puromycin for 4 h prior to cell harvest to prevent the nonsense-mediated RNA decay [51]. Details on cell culture, RNA-extraction and cDNA synthesis can be found in [37]. BigDye Terminator Cycle Sequencing chemistry was used for sequencing (Applied Biosystems, Foster City, CA). The sequencing reactions were subsequently run on an automated capillary sequencer and analyzed using the sequence analysis program SeqScape v2.5 (Applied Biosystems, Foster City, CA) and/or SequencePilot (JSI Medical Systems, Kippenheim, Germany). In some instances it was necessary to manually analyze (read) the aberrant sequences in order to determine all splicing defects deducible from multiple overlaying sequences (see Figure S1).

*NF1* nucleotide numbering is based on GenBank reference sequence NM_000267.3 with the A of the ATG start codon being nucleotide position c.1. The integration sites are given according to the HGVS nomenclature after the first duplicated sequence regardless of whether the first nick occurred at the sense or the anti-sense strand and the thereof resulting orientation of the insertion with respect to the *NF1* coding sequence. Exons are numbered according to the reference sequence with the widely known legacy numbering given in parenthesis.

### Identification and confirmation of L1 EN-mediated insertions

Exons and flanking intronic sequences affected by splicing defects were analyzed by sequencing from genomic DNA and by multiplex ligation dependent probe amplification (MLPA) with the current SALSA-MLPA-kit P081-B1 and/or P082-B1 (MRC-Holland, Amsterdam, the Netherlands). When MLPA results were negative for a genomic deletion of the respective exon and no alteration was identified explaining the observed splicing defect, sequences were meticulously reanalyzed to uncover a possible *Alu*/L1 insertion within or in close vicinity of the exon (see Figure S2). To diminish allelic drop-out of the mutant allele due to the increased exon size by an *Alu*/L1 insertion, the PCR conditions were modified to allow for the amplification of larger sequences from gDNA of the patients. Primers and PCR conditions used to amplify the affected exons are listed in Table S1A and S1B. Mutant exons containing *Alu* elements were amplified using Takara Ex Taq (Takara Biotechnology Co. LTD, Madison, WI) or *Taq* DNA Polymerase (Invitrogen, Carlsbad, CA). To amplify the mutant alleles containing a truncated 1753-bp and a full-length 6-kb L1 element, respectively, Expand Long PCR Taq (Roche Diagnostics GmbH, Roche Applied Science, Mannheim, Germany) was used. To determine the precise sequence of the inserted *Alu* element as well as the duplicated sequence at the insertion site the PCR products showing a band of increased size were cloned according to the manufacturer's instructions into the TOPO-TA cloning vector pCR 4-TOPO (Invitrogen, Carlsbad, CA) and individually sequenced. Alternatively or in addition, primers were designed to specifically amplify only the mutant alleles containing the *Alu* insertions. These primers contained at their 5′-end the exonic sequences immediately upstream/downstream of the insertion site and at the 3′-end a few nucleotides of either the *Alu* or the poly(A/T) stretch. All primers are listed in Table S1A. PCR products generated with these primers used together with the regular exon primer at the opposite site of the exon were subsequently sequenced (see as an example Figure S3). Alignment of the identified *Alu* sequences with the consensus sequences of the different *Alu* families (as deposited in Repbase Giri [29]) was performed with the program ClustalW v.1.83 [58]. To amplify the much larger mutant alleles containing a truncated 1753-bp and a full-length 6-kb L1 element, the Expand Long PCR Taq kit (Roche Diagnostics GmbH, Roche Applied Science, Mannheim, Germany) specifically designed to amplify fragments up to around 20 kb, was used. To sequence the 5′-end of a full length L1 element integrated in exon 39 (30) a L1-specific reverse primer (L1-5_39r in Table S1) was used together with the exon 39 (30) forward primer (39f). Thereafter, the entire 6021-bp L1 sequence was characterized by sequencing of the PCR product generated with primers 39f and 39r (Figure 2) using L1-sequence specific internal primers (Table S1). Similarly, to amplify a mutant 1800-bp fragment containing a truncated L1 element in exon 23 (18), and thereafter sequence the 5′ and 3′ends of this mutant fragment, we used the primers 23f and 23r. In addition, sequence analysis was performed with a L1-specific primer, L1_Fam (Table S1), located 86 bp upstream of a 3-bp diagnostic site that distinguishes the young L1 subfamilies (Ta) and pre-(Ta) from the older ones [35]. To determine the sequence inserted into intron 9 (7) that lead, at the transcript level, to loss of exon 9 (7) and concomitant insertion of an L1-derived 130-bp sequence between exons 8 (6) and 10 (8) in patient UAB-R91409, a reverse and a forward primer (Line9_r and Line10_f in Table S1) were used in two PCR reactions together with the exon 9 (7) forward (9f) and the exon 10 (8) reverse (10r) primer, respectively. The resulting PCR products were sequenced in both directions.

## Supporting Information

Figure S1 Detection of two aberrant splice products due to an *Alu*Ya5 insertion in exon 47 (38). The sequence generated with a reverse primer from a RT-PCR product from the patient shows the border of *NF1* exons 47 (38) and 48 (39). In addition to the wild type transcript two aberrantly spliced transcripts can be deduced from the sequence. One aberrant transcript lacks the entire exon 47 (38) and the other the last 62 nucleotides of exon 47 (38) due to the use of an exonic cryptic 5′-splice site upstream of the integration site.
(TIF)

Figure S2 Detection of an *Alu*Ya5 insertion in *NF1* exon 47 (38). A) Agarose gel showing PCR products generated from gDNA from a control individual (C) and the patient (P) harboring an *Alu*Ya5 insertion in *NF1* exon 47 (38), (W = water, M = size Marker). PCR product of the patient shows a faint extra band of larger size (arrow) that is not present in the control. B) Sequences of the PCR products from the control individual and the patient. The sequence of the patient shows a faint background sequence (a poly(T) stretch) starting at nucleotide c.6952 (vertical dotted line). This indicates the insertion of a retrotransposon, in this case an *Alu*Ya5 element, in anti-sense direction with respect to the *NF1* coding sequence at this site.
(TIF)

Figure S3 Specific PCR for the *Alu*Ya5 insertion in *NF1* exon 47 (38). A) Scheme showing the strategy to amplify the *Alu*Ya5 insertion. Exons are shown as boxes and introns as lines. The *Alu*-insertion is shown as an arrow in anti-sense orientation. In order to specifically amplify the mutant allele containing the *Alu* sequence two *Alu* insertion specific primers (Alu_47f and Alu_47r) spanning the exon 47 (38)-*Alu* insertion border were designed. These primers were used together with their respective regular exon primer (47r or 47f) at the opposite site of the exon resulting in fragments of 600 bp and 1000 bp, respectively, each containing the *Alu* insertion. C) Agarose gel showing the result of the *Alu*-insertion specific PCRs derived from the patient (P) and a control individual (C). B) Sequence analysis of the specific *Alu*-insertion product generated by the *Alu* insertion-specific reversed primer and the respective regular forward primer, shows the anti-sense orientation of the *Alu* insertion.
(TIF)

Figure S4 Sequence alignment of the 14 *Alu* sequences with the consensus sequence of the *Alu*Y, *Alu*Ya5 and *Alu*Yb8 subfamilies. Identical nucleotides between all sequences are indicated by capital letters. In order to maximize the alignment gaps were introduced (dashed). The reference sequences of the *Alu* subfamilies were taken from Repbase Giri [29].
(TIF)

Figure S5 Alignment of the full length L1 sequence with the consensus sequence of the hot L1 element. The element was found inserted in sense orientation into the *NF1* exon 39 (30) of a patient (R01429). The sequence of the hot L1 element is taken from [2]. Ten deviations from the consensus sequence, two of which in the ORF1 and five in ORF2 (six of them altering the amino acid code) are highlighted by light background.
(TIF)

Figure S6 Truncated L1 element inserted into intron 9 (7). Genomic sequence of intron (IVS) 9 (7) (black letters) with truncated L1 element (green letters) inserted at position c.1062+195_1062+196 and the flanking exons 9 (7) and 10 (8) (red letters). The inserted sequence contains at the 5′-end a poly(T) stretch indicating that the poly(A) tail of the L1 transcript had annealed to the sense strand where reverse transcription from the template started. However, the 3′-end of the inserted sequence contains in sense orientation with regard to the *NF1* coding sequence at least 1088 bp from the center of the L1 ORF2 and ends with the 3167^th^ nucleotide of the 3825-bp L1 ORF2 suggesting that during the process of retrotransposition the orientation of the reverse transcription from the L1-RNA template that started at the sense strand of the *NF1* gene switched and continued from the anti-sense strand. The duplicated nucleotides of the TSD are underlined. The 130-bp cryptic exon embedded in the L1 element is indicated in a darker shade of green. The sequence used to design L1 insertion-specific primers is underlined. The splices site scores of the cryptic exon as calculated by Splice Site Prediction by Neural Net (http://www.fruitfly.org/seq_tools/splice.html) is 0.62 for the 5′ splice site and 0.86 for the 3′ splice site.
(TIF)

Table S1 (A) List of primers for amplification of *NF1* exons, *Alu* and L1 sequences. (B) List of PCR programs.
(PDF)
