# Supplementary figures and images for: The NF1 Gene Contains Hotspots for L1 Endonuclease-Dependent De Novo Insertion
Source: PLoS Genet. 2011 Nov 17;7(11):e1002371. doi: 10.1371/journal.pgen.1002371 (PMC3219598; doi:10.1371/journal.pgen.1002371)

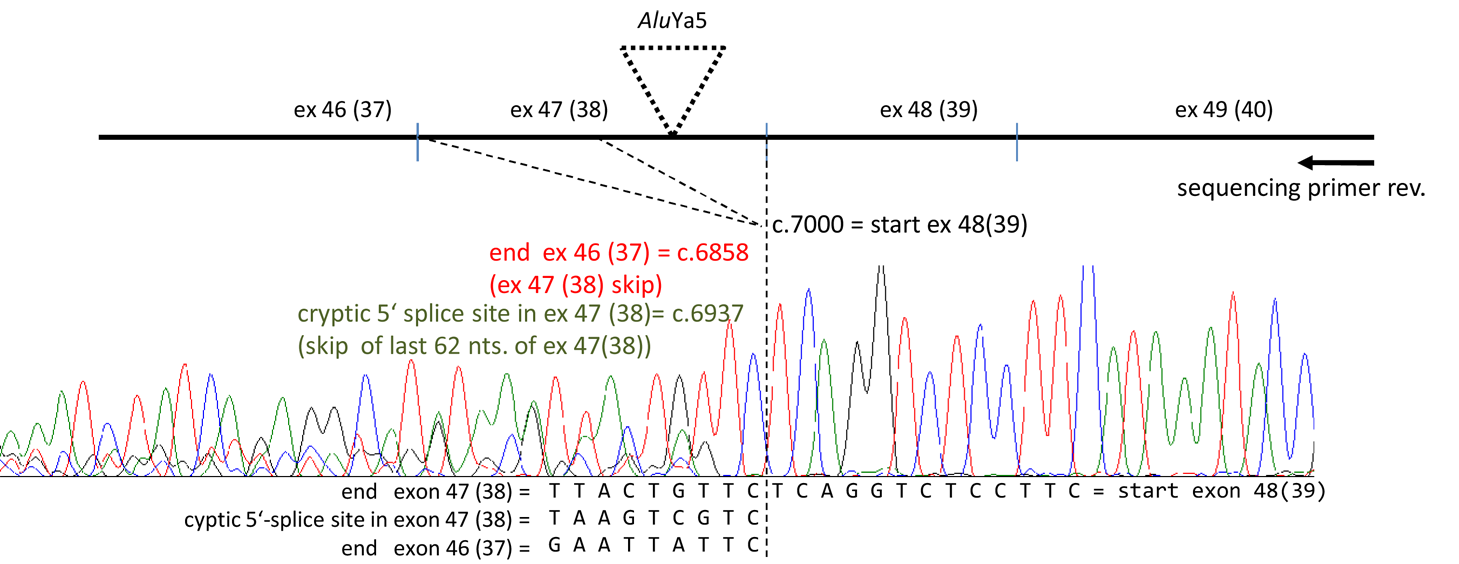

Supplement: Figure S1 — Detection of two aberrant splice products due to an AluYa5 insertion in exon 47 (38). The sequence generated with a reverse primer from a RT-PCR product from the patient shows the border of NF1 exons 47 (38) and 48 (39). In addition to the wild type transcript two aberrantly spliced transcripts can be deduced from the sequence. One aberrant transcript lacks the entire exon 47 (38) and the other the last 62 nucleotides of exon 47 (38) due to the use of an exonic cryptic 5′-splice site upstream of the integration site. (TIF) [file pgen.1002371.s001.tif]

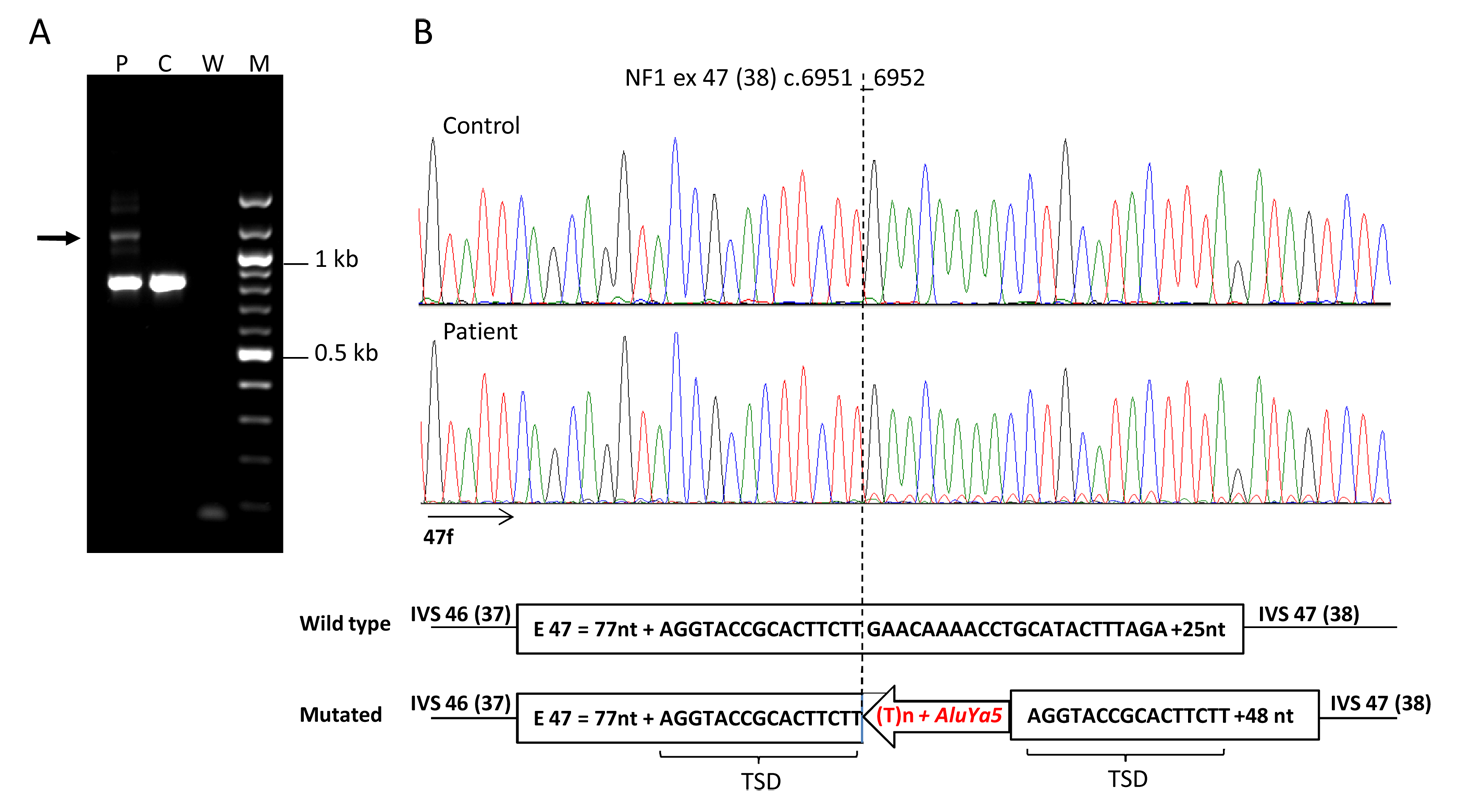

Supplement: Figure S2 — Detection of an AluYa5 insertion in NF1 exon 47 (38). A) Agarose gel showing PCR products generated from gDNA from a control individual (C) and the patient (P) harboring an AluYa5 insertion in NF1 exon 47 (38), (W = water, M = size Marker). PCR product of the patient shows a faint extra band of larger size (arrow) that is not present in the control. B) Sequences of the PCR products from the control individual and the patient. The sequence of the patient shows a faint background sequence (a poly(T) stretch) starting at nucleotide c.6952 (vertical dotted line). This indicates the insertion of a retrotransposon, in this case an AluYa5 element, in anti-sense direction with respect to the NF1 coding sequence at this site. (TIF) [file pgen.1002371.s002.tif]

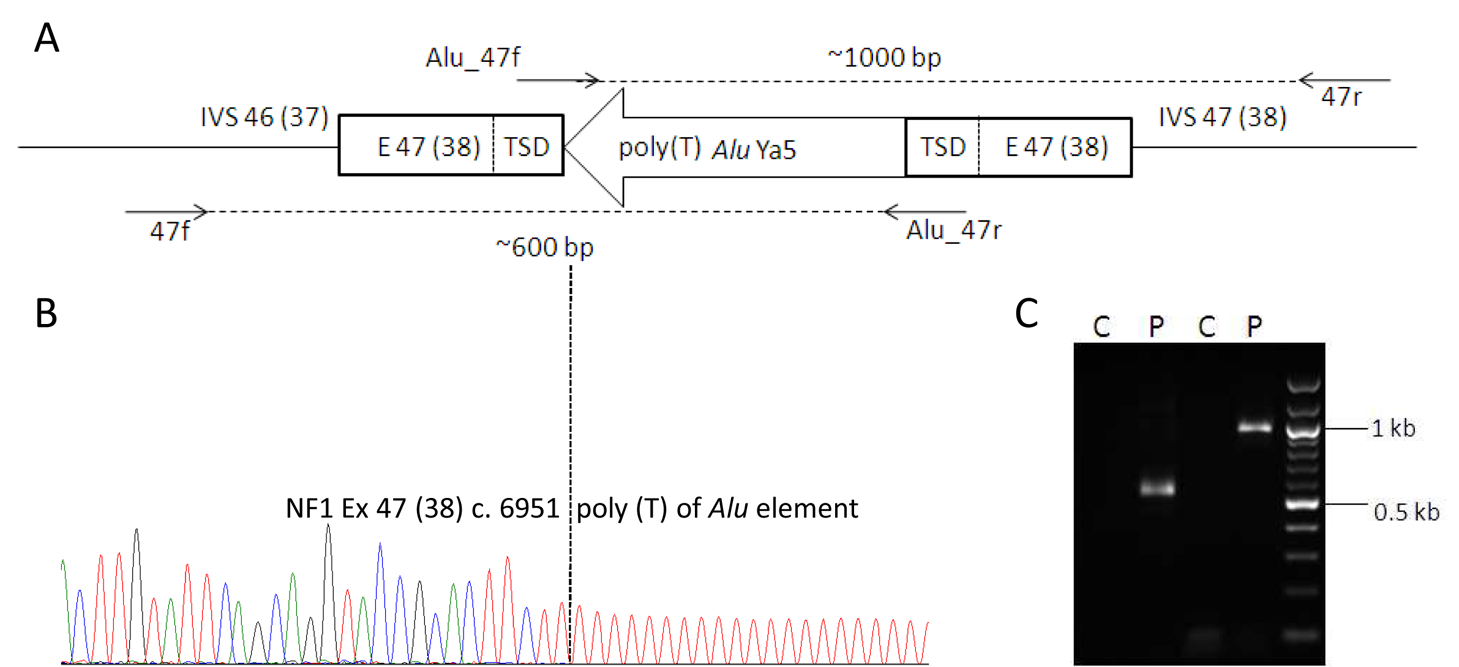

Supplement: Figure S3 — Specific PCR for the AluYa5 insertion in NF1 exon 47 (38). A) Scheme showing the strategy to amplify the AluYa5 insertion. Exons are shown as boxes and introns as lines. The Alu-insertion is shown as an arrow in anti-sense orientation. In order to specifically amplify the mutant allele containing the Alu sequence two Alu insertion specific primers (Alu_47f and Alu_47r) spanning the exon 47 (38)-Alu insertion border were designed. These primers were used together with their respective regular exon primer (47r or 47f) at the opposite site of the exon resulting in fragments of 600 bp and 1000 bp, respectively, each containing the Alu insertion. C) Agarose gel showing the result of the Alu-insertion specific PCRs derived from the patient (P) and a control individual (C). B) Sequence analysis of the specific Alu-insertion product generated by the Alu insertion-specific reversed primer and the respective regular forward primer, shows the anti-sense orientation of the Alu insertion. (TIF) [file pgen.1002371.s003.tif]

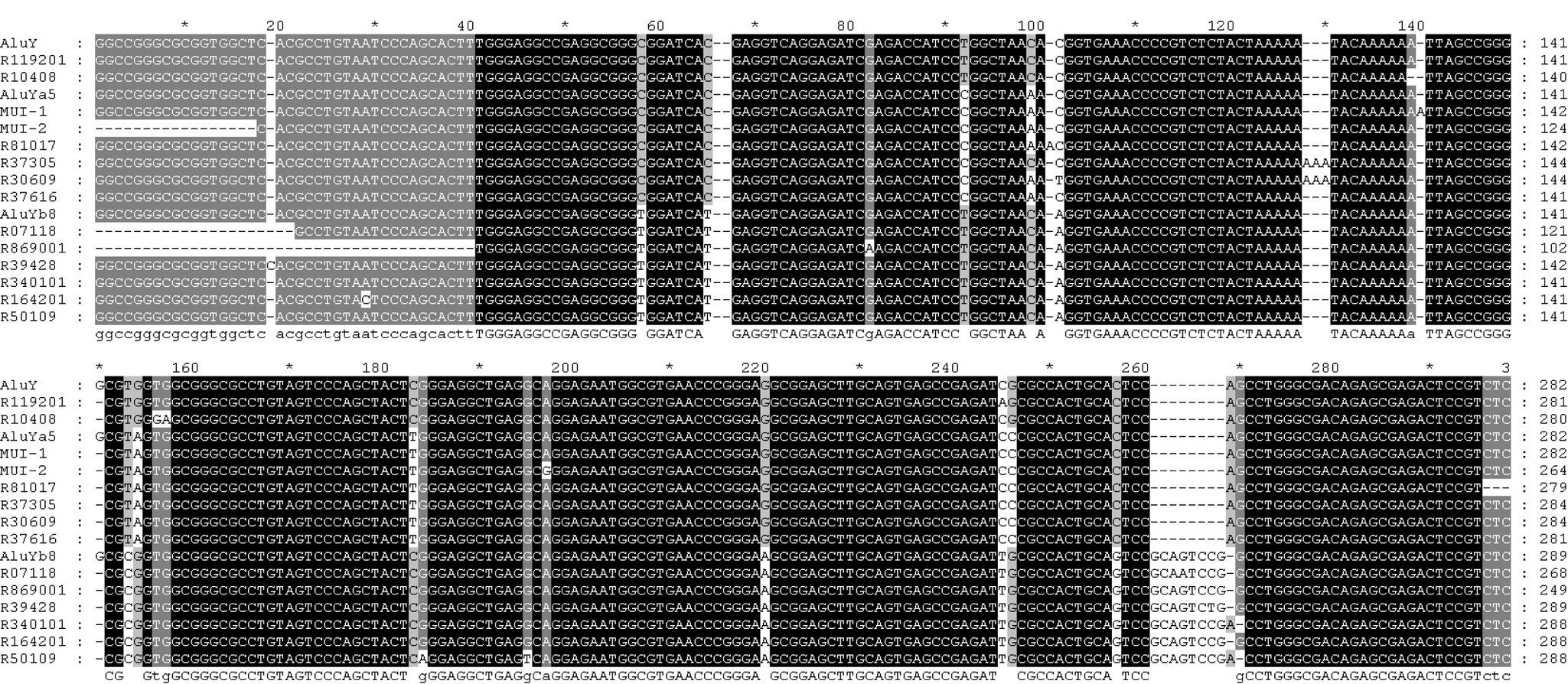

Supplement: Figure S4 — Sequence alignment of the 14 Alu sequences with the consensus sequence of the AluY, AluYa5 and AluYb8 subfamilies. Identical nucleotides between all sequences are indicated by capital letters. In order to maximize the alignment gaps were introduced (dashed). The reference sequences of the Alu subfamilies were taken from Repbase Giri [29]. (TIF) [file pgen.1002371.s004.tif]

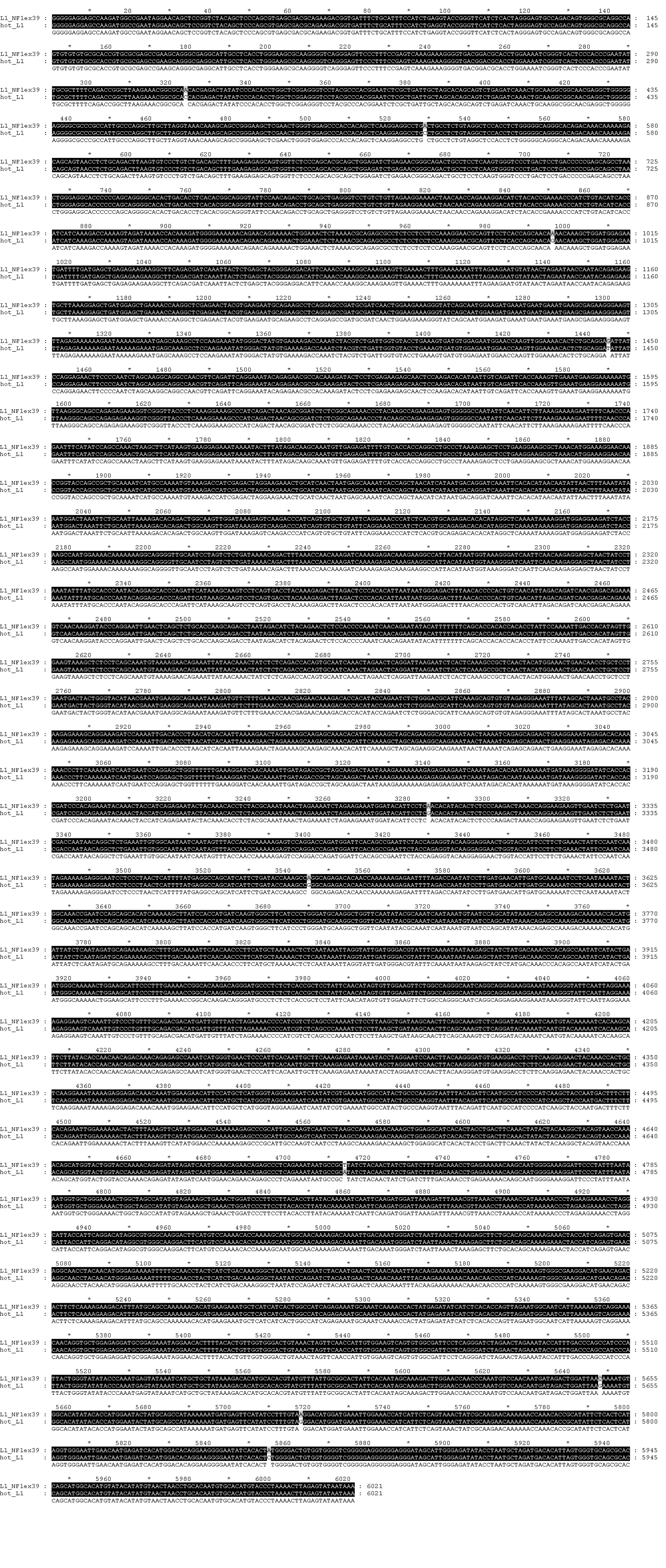

Supplement: Figure S5 — Alignment of the full length L1 sequence with the consensus sequence of the hot L1 element. The element was found inserted in sense orientation into the NF1 exon 39 (30) of a patient (R01429). The sequence of the hot L1 element is taken from [2]. Ten deviations from the consensus sequence, two of which in the ORF1 and five in ORF2 (six of them altering the amino acid code) are highlighted by light background. (TIF) [file pgen.1002371.s005.tif]

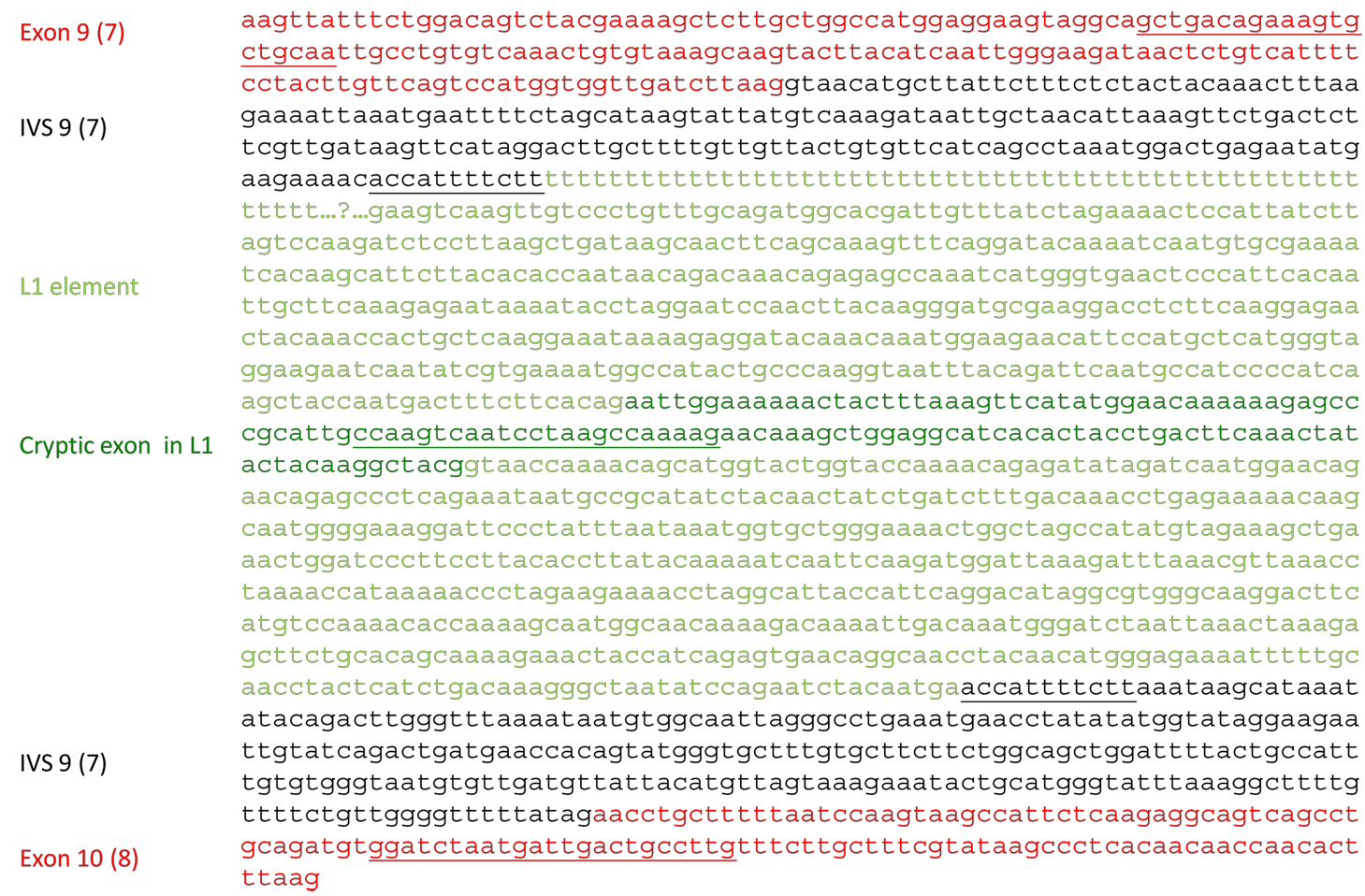

Supplement: Figure S6 — Truncated L1 element inserted into intron 9 (7). Genomic sequence of intron (IVS) 9 (7) (black letters) with truncated L1 element (green letters) inserted at position c.1062+195_1062+196 and the flanking exons 9 (7) and 10 (8) (red letters). The inserted sequence contains at the 5′-end a poly(T) stretch indicating that the poly(A) tail of the L1 transcript had annealed to the sense strand where reverse transcription from the template started. However, the 3′-end of the inserted sequence contains in sense orientation with regard to the NF1 coding sequence at least 1088 bp from the center of the L1 ORF2 and ends with the 3167th nucleotide of the 3825-bp L1 ORF2 suggesting that during the process of retrotransposition the orientation of the reverse transcription from the L1-RNA template that started at the sense strand of the NF1 gene switched and continued from the anti-sense strand. The duplicated nucleotides of the TSD are underlined. The 130-bp cryptic exon embedded in the L1 element is indicated in a darker shade of green. The sequence used to design L1 insertion-specific primers is underlined. The splices site scores of the cryptic exon as calculated by Splice Site Prediction by Neural Net (http://www.fruitfly.org/seq_tools/splice.html) is 0.62 for the 5′ splice site and 0.86 for the 3′ splice site. (TIF) [file pgen.1002371.s006.tif]
