# Supplementary material for: The NF1 Gene Contains Hotspots for L1 Endonuclease-Dependent De Novo Insertion
Source: PLoS Genet. 2011 Nov 17;7(11):e1002371. doi: 10.1371/journal.pgen.1002371 (PMC3219598; doi:10.1371/journal.pgen.1002371)
Supplement: Table S1 — (A) List of primers for amplification of NF1 exons, Alu and L1 sequences. (B) List of PCR programs. (PDF) [file pgen.1002371.s007.pdf]

Table S1 A. List of primers for amplification of *NF1* exons, *ALU* - and L1 sequences

Exon	Name	Sequence 5'-3'	Orientation	Size	PCR	Enzyme	Buffer
6 (4c)	6cf	GCAAAAGTAATACGTAAATGGAAAG	fwd	904/>1170	1	Invitrogen	
	6cr	TGATGTACCCAAGCAACAAAGAC	rev				
6 (4c)	6cf	GCAAAAGTAATACGTAAATGGAAAG	fwd	403/- *	2	Invitrogen	
	6c_sr	CTACCCAGTTCCAAAATGCC	rev				
11 (9)	11f	CTTTCTATTTGCTGTTCTTTTTGG	fwd	264/-§	6	Roche	1,2&3
	11f_new	TATTTCTCACTATTATGTATTGATGTTCGT	fwd	330/>550			
	11r	CCTTTTTGAAAACCAAGAGTGCAT	rev				
12 (10a)	12af	ACGTAATTTTGTACTTTTTCTTCC	fwd	222/>500	3	Platinum	
	12ar	CAATAGAAAGGAGGTGAGATTC	rev				
15(11)	15f	CTCCAGTGTTATGTTTACCAAAAA	fwd	263/>600	4	Takara	
	15r	CTTTGGAAGTGTAAGTTTTACAATGT	rev				
21 (16)	21f	GGAAGAAATGTTGGATAAAGCA	fwd	610/>1000	4	Takara	
	21r	TTAAATAATCTGAAAGAAAAAGGCTTA	rev				
22 (17)	22f	CTCTGTGTGTTTAGATCAGTCA	fwd	319/>700	4	Platinum	
	22r	TTTATCAATTACTACCAGTATCAG	rev				
23 (18)	23f	AGAAGTTGTGTACGTTCTTTTCT	fwd	367/>2000	6	Roche	3
	23r	CTCCTTTCTACCAATAACCGC	rev				
25 (19b)	25f	ATTACCTTCTCCCCATTTGA	fwd	372/~500	4	Takara	
	25r	GGCTTTATTTGCTTTTTGC	rev				
33 (25)	33f	CATTTTATTATAGCAGATGTC	fwd	533/>900	5	Takara	
	33r	ACTTACACAGGAACTTCAT	rev				
39 (30)	39f	TTGGAACTATAAGGAAAAATACGTTT	fwd	321/>6000?	6		
	39r	AGGGTTTTCTTTGAATTCTCTTAGA	rev				
47 (38)	47f	ATAGACCTCATCTCACATTCAGC	fwd	816/>1080	2	Invitrogen	
	47r	CATAGTTCTTTATGAGCTGGTAAGG	rev				
49 (40)	49f	CCTTTCCTTGCAGAGTTGTTA	fwd	371/>700	4	Takara	
	49r	CACCACTAAAGGACTAGACTGT	rev				
***Alu* - and LINE specific PCR primer**							
6 (4c)	Alu_6cf	AGTTATAAATAGCCTGGAGGCC	fwd	~ 1200	7	Invitrogen	
	6cr	TGATGTACCCAAGCAACAAAGAC	rev				
9 (7)	9f	GCTGACAGAAAGTGCTGCAA	fwd	~1300bp	6	Roche	1,2&3
	Line9_r	CTTTTGGCTTAGGATTGACTTGG	rev				
	Line10_f	CCAAGTCAATCCTAAGCCAAAAG	fwd	934	4	Platinum	
	10r	CAAGGCAGTCAATCATTAGATCC	rev				
39 (30)	39f	GTAACAGAATCACAAATTGTATGTTA	fwd	355	8	Platinum	
	L1-5_39r	CTTCCCAGGTGAGGCAATG	rev				
47 (38)	Alu_47f	GCAGGTACCGCACTTCTTTT	fwd	~ 600	7	Invitrogen	
	47r	CATAGTTCTTTATGAGCTGGTAAGG	rev				
	47f	ATAGACCTCATCTCACATTCAGC	fwd	~ 950	7		
	Alu_47r	CAAGAAGTGCGGTACCTCAC	rev				
Line_Fam	L1_Fam	GAACAATGAGATCACATGGACAC	fwd	Sequencing			
L1 (~6kb)	Line_2f	AAGCAGGGCGAGGCATTG	fwd	Sequencing			
	Line_3f	CAGACTGCCTCCTCAAGTG	fwd	Sequencing			
	Line_4f	CAAGAAGTGCGGTACCTCAC	fwd	Sequencing			
	Line_5f	GAAACCCTACAAGCCAGAAG	fwd	Sequencing			
	Line_6f	AGACACAGACTGGCAAGTTG	fwd	Sequencing			
	Line_7f	GCACCAAGCAGACCTAATAGAC	fwd	Sequencing			
	Line_8f	TGCCTACAAGAGAAAGCAGG	fwd	Sequencing			
	Line_2r	GCTGCACCCACTAATGTGTC	rev	Sequencing			
	Line_3r	GCCATTCTAACTGGTGTGAG	rev	Sequencing			
	Line_4r	GCTTTCTACATATGGCTAGCC	rev	Sequencing			
	Line_5r	AGTTCTCCTTGAAGAGGTCC	rev	Sequencing			
	Line_6r	GAGGGTTTGTCATAGATAGCTC	rev	Sequencing			

wt = wild type allele, mut = mutant allele, program = PCR program, fwd = forward primer, rev = reverse primer, * mutant allele does not amplify under these conditions; § does not amplify the mutant allele since the primer is within the deleted sequence associated with the *Alu* insertion; primer 11f_new was used to amplify the mutant allele

Table S1 B. List of PCR programs

Prog. nr.	Denat.	Cycle 1	Cycle 2	Final elong.	Store
1	95° 3‘	(94° 30‘‘ - 59° 30‘‘ - 72° 4‘) x 40	-	72° 7‘	4°
2	95° 3‘	(94° 20‘‘ - 59.5° 25‘‘ - 72° 45‘‘) x 40	-	72° 7‘	4°
3	94° 3‘	(94° 20‘‘ - 61° 15‘‘(-1°/cycle) - 72° 1‘) x 11	(94° 40‘‘ - 52° 40‘‘ - 72° 30‘‘) x 24	72° 10‘	15°
4	92° 4‘	(92° 30‘‘ - 58° 30‘‘ - 72° 3‘) x 19	(92° 30‘‘ - 58° 30‘‘ - 72° 3‘ (+10‘‘/cycle)) x 25	72° 10‘	15°
5	95° 5‘	(95° 30‘‘ - 58° 20‘‘ - 72° 1‘30‘‘) x 35	-	72° 10‘	15°
6	94° 2‘	(94° 10‘‘ - 58° 30‘‘ - 68° 10‘) x 10	(94° 15‘‘ - 58° 30‘‘ - 68° 10‘(+20‘‘/cycle)) x 25	68° 7‘	15°
7	95° 3‘	(94° 20‘‘ - 62° 20‘‘ - 72° 1‘) x 10	(94° 40‘‘ - 52° 40‘‘ - 72° 1‘) x 30	72° 10‘	4°
8	95° 3‘	(95° 20‘‘- 65° 15‘ ‘(- 1°/cycle) - 72° 30‘‘)x8	(95° 30‘‘ - 58° 30‘‘ - 72° 30‘‘) x 25	72° 10‘	15°
